# Predictors of non-suicidal self-injury and moderating effects of cognitive emotion regulation strategies in Korean undergraduate students: Secondary data analysis of a cross-sectional survey

**DOI:** 10.1371/journal.pone.0344175

**Published:** 2026-03-20

**Authors:** Yeo Won Jeong

**Affiliations:** Department of Nursing, College of Nursing, Dongguk University-WISE, Gyeongju, Korea; University of Botswana, BOTSWANA

## Abstract

**Background:**

Non-suicidal self-injury (NSSI) is an emerging mental health issue in both clinical practice and public health worldwide, refers to intentional self-harm without suicidal intent. Exploring protective and moderating factors of NSSI is important for its prevention and intervention. However, most existing studies have focused primarily on risk factors. This study aimed to identify predictors of NSSI and examine the moderating effects of cognitive emotion regulation strategies on the relationship between psychological discomfort and NSSI.

**Methods:**

This study involved a secondary analysis of data from 188 self-reported questionnaires collected via a web-based survey in South Korea between August and September 2020 during COVID-19 pandemic. NSSI was measured using the modified NSSI subscale of the Korean version of the Self-Harm Inventory. Psychological discomfort was assessed using a 20-item symptom checklist, and cognitive emotional regulation strategies were measured using the Cognitive Emotion Regulation Questionnaire. Data were analyzed using zero-inflated negative binomial regression. The Hayes PROCESS macro (Model 1) was used to examine moderating effects.

**Results:**

Of the 188 participants, 159 were female. A total of 30.3% reported experiencing NSSI within the past six months. NSSI was associated with being female and lower use of positive cognitive emotion regulation strategies. Psychological discomfort was positively associated with NSSI. Positive cognitive emotion regulation strategies had a conditional moderating effect on the relationship between psychological discomfort and NSSI. However, negative cognitive emotion regulation strategies did not show a significant moderating effect.

**Conclusions:**

Psychological discomfort is a salient risk factor for NSSI. Positive cognitive emotion regulation strategies may be an important protective factor. Therefore, prevention and intervention efforts should emphasize strengthening positive—rather than negative—cognitive emotion regulation strategies. To reduce NSSI among undergraduates with a history of NSSI, policies or psychoeducation should focus on increasing positive cognitive coping. Moreover, tailored interventions are needed to reduce psychological discomfort among students with no history of NSSI.

## Introduction

Non-suicidal self-injury (NSSI) refers to the direct and intentional act of harming one’s own body without suicidal intent [1] and is an emerging mental health concern in both clinical practice and public health worldwide. After adolescence, NSSI is the second most prevalent among individuals in their 20s [2]. Valencia reported a prevalence rate of 38.9% [3]. Among undergraduate students, various stressful life events, such as academic pressure, adjustment to new environments [4,5], interpersonal strife [2], and bullying [6], are associated with mental health issues, including depression and anxiety [4,5,7]. The psychological discomfort associated with these conditions has been shown to increase the likelihood of NSSI [3]. Previous studies have identified depressive symptoms, hopelessness, anxiety, and eating disorders as risk factors for NSSI among college students [2,8,9].

Both traumatic or stressful experiences and psychological discomfort are risk factors for NSSI [2,10]; however, it is possible that traumatic or stressful experiences contribute to NSSI by increasing psychological discomfort. Psychological discomfort refers to negative emotional reactions, such as depression, anxiety, and somatization, that individuals experience in certain situations [11,12]. Previous studies have found that traumatic experiences elevate psychological distress [13] and that such discomfort increases the risk of NSSI [3,10]. The coronavirus disease 2019 (COVID-19) pandemic has also been identified as a traumatic event [14], elevating college students’ anxiety, depression [15,16], and NSSI [17]. While these studies provided valuable insights into the association between specific variables and NSSI, they did not address the overall psychological discomfort experienced in special situations such as the COVID-19 pandemic and NSSI. Some studies have reported that psychological discomfort during the pandemic was significantly higher in nature compared to normal times [18–20], therefore, it is necessary to investigate whether psychological discomfort due to traumatic or stressful life experiences, including the pandemic, affects the incidence of NSSI among college students during this transitional period.

Understanding the protective and moderating factors of NSSI is crucial for prevention and intervention. Previously reported moderating factors include self-concept [3] and self-esteem [21], while protective factors include meaning of life and life satisfaction [22], adaptive coping strategies [23], and emotion regulation strategies [24]. Cognitive emotion regulation strategies (CERS) refer to how individuals manage or regulate emotions in response to threatening or stressful events [25]. These strategies can serve as protective factors, risk factors, or moderators. Garnefski and Kraaij describe CERS as comprising nine dimensions, which are categorized as either positive-focused CERS (PCERS)—theoretically more adaptive (e.g., acceptance, putting into perspective, positive refocusing, refocus on planning, and positive reappraisal)—or negative-focused CERS (NCERS)—theoretically less adaptive (e.g., self-blame, catastrophizing, blaming others, and rumination) [25]. The definition of these nine CERS dimensions, as described by Garnefski, Kraaij and Spinhoven (2001) [25], are as follows: “*Acceptance* refers to thoughts of accepting what you have experienced and resigning yourself to what has happened; *Putting into perspective* refers to thoughts of playing down the seriousness of the event or emphasizing its relativity when compared to other events; *Positive refocusing* refers to thinking about joyful and pleasant issues instead of thinking about the actual event; *Refocus on planning* refers to thinking about what steps to take and how to handle the negative event; *Positive reappraisal* refers to thoughts of attaching a positive meaning to the event in terms of personal growth; *Self-blame* refers to thoughts of blaming yourself for your experience; *Catastrophizing* refers to thoughts of explicitly emphasizing the terror of an experience; *Blaming others* refers to thoughts of putting the blame of what you have experienced on others; *Rumination* refers to thinking about the feelings and thoughts associated with the negative event” (pp. 1314–1316). Cognitive positive reappraisal has been shown to reduce NSSI severity [24], while rumination increases it [24]. Furthermore, CERS plays a crucial role in the relationship between negative life experiences and symptoms of depression and anxiety [25]. However, previous studies have generally focused on risk factors for NSSI, paying limited attention to protective factors, mediators, and moderators [3]. Continued research into these mediating and moderating variables is needed to better understand the complex relationships between NSSI and its predictors. Furthermore, past studies have often measured CERS only partially [26] and have not systematically explained the moderating effects of PCERS and NCERS on NSSI.

Recent research has reported a second peak in NSSI occurrence around age 20 [27]. Given that NSSI is a known risk factor for suicide, it is necessary to identify both protective and risk factors to improve mental health outcomes within the community.

**Fig 1:**
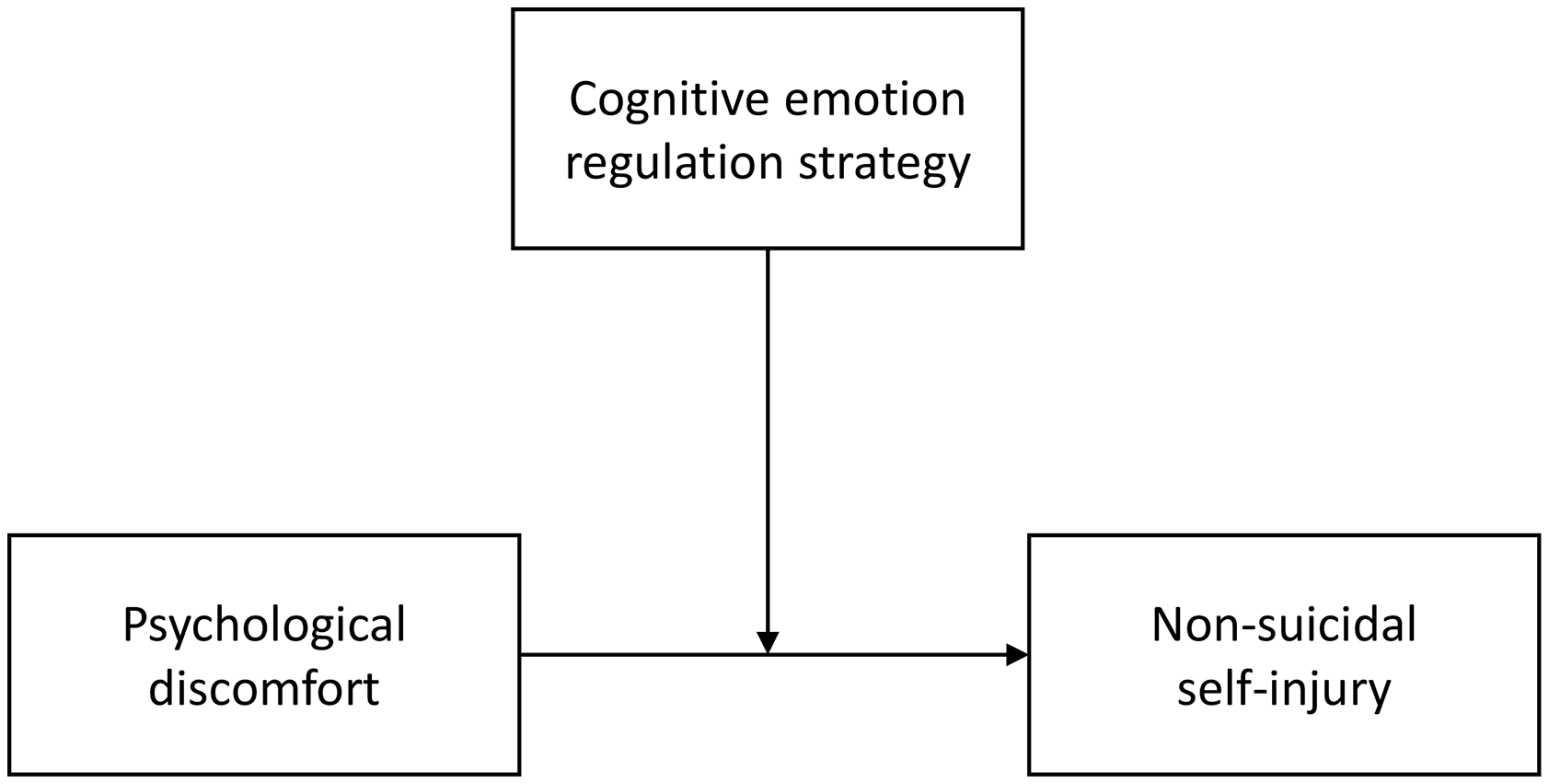
Hypothesized model of the study.

In this context, this secondary data analysis used survey data [28] to explore NSSI predictors among undergraduate students and verify the moderating effect of CERS on the relationship between psychological discomfort and NSSI. The detailed objectives of this study were to 1) examine the levels of NSSI, psychological discomfort, and CERS; 2) identify predictors of NSSI; and 3) examine the moderating effect of CERS on the relationship between psychological discomfort and NSSI among undergraduate students (Fig 1). The proposed hypotheses are as follows:

1) Hypothesis 1 (H1): Psychological discomfort will be positively associated with NSSI.
2) Hypothesis 2 (H2): CERS will moderate the relationship between psychological discomfort and NSSI.

## Methods

### Participants

The participants included 188 undergraduate students recruited through a web-based survey using convenience sampling, rather than in-person surveys. In-person surveys related to suicide or NSSI might not elicit honest responses due to participants’ fear of compromising social desirability [29]. The inclusion criteria were currently being enrolled in years 1–4 at a university or college, or taking a leave of absence. Among the participants, 159 (84.6%) were female, and the mean age was 20.61 (standard deviation [SD] = 1.82; range = 18–29 years). The required sample size of the study was calculated using G*Power 3.1.9.2, Poisson regression [30], considering a significant level of.05, a study power of 85%, and a dropout rate of 15%.

### Measures

#### 1) NSSI.

Participants’ NSSI history over the past six months was measured using the Korean version [31] of the Self-Harm Inventory (K-SHI), originally developed by Sansone et al. (1998) [32]. The NSSI subscale assesses five types of lifetime NSSI behaviors: cutting, burning, hitting, head-banging, and scratching [33]. Previous studies have also reported behaviors such as overdosing or engaging in sexual risk-taking as self-damaging, even if they do not involve intentional tissue damage, and have conceptualized or categorized them as NSSI [34,35]. Therefore, the scale was modified by adding five additional items: overdose (one item); abusive relationships (three items); and preventing wounds from healing (one item). Each item included the phrase “without the intention of killing yourself.” Items were rated as yes/no (0 = no; 1 = yes), and total scores ranged from 0 to 22, with higher scores indicating greater NSSI behavior. During analysis, participants who answered “yes” to any K-SHI item (i.e., scored 1 or more) were classified into the NSSI group; those who answered “no” to all items (score of 0) were classified into the non-NSSI group. The reliability of the Korean version of the scale at the time of development was KR-20 = .76 [31]; in this study, it was.776.

#### 2) Psychological discomfort.

Psychological discomfort was assessed using the 20-item Symptom Checklist [11], which comprises four subcategories: depression/withdrawal, hyperalertness, generalized anxiety, and somatic complaints. Items are rated on a 4-point Likert scale ranging from 0 (“not at all”) to 3 (“very often”), in response to the question: “How many times in the past few weeks did you experience the symptom?” Examples of symptoms include “depressed mood,” “difficulty concentrating,” “worry,” and “upset stomach.” Total scores range from 0 to 60, with higher scores indicating greater psychological discomfort, typically associated with greater exposure to trauma [11]. Although originally developed for military populations [11], this scale has also been used with adults with borderline personality disorder [36], the general population [37], and university students [12]. The Korean version was used in this study [12]. At the time of development, Cronbach’s alpha ranged from.90 to.93, and was.88 for the Korean version [12]; in this study, it was.896.

#### 3) Cognitive emotion regulation strategies.

The cognitive emotion regulation questionnaire (CERQ), developed by Garnefski, Kraaija, and Spinhoven [25], was used to assess participants’ CERS. The Korean version (K-CERQ), validated by Ahn, Lee, and Joo, was used in this study [38]. The K-CERQ is a reliable and valid self-report questionnaire for identifying individual differences in CERS following negative events [38]. Previous studies have demonstrated the CERQ’s usefulness in measuring CERS in individuals with NSSI who have experienced threatening or stressful events [26,39,40]. The questionnaire comprises 35 items across two categories and nine factors: five PCERS (acceptance, putting into perspective, positive refocusing, refocusing on planning, and positive reappraisal), and four NCERS (self-blame, catastrophizing, blaming others, and rumination) [41]. Each factor, except “acceptance” (three items), comprises four items. All are rated on a 5-point Likert scale (1 = almost never; 5 = almost always). Example items include “I think that I have to accept that this has happened (acceptance),” “I think that it hasn’t been too bad compared to other things (putting into perspective),” “I think I can learn something from the situation (positive reappraisal),” “I think that basically the cause must lie within myself (self-blame),” “I often think that what I have experienced is much worse than what others have experienced (catastrophizing),” “I dwell upon the feelings the situation has evoked in me (rumination).” Higher scores indicate higher use of the CERS. At the time of development, Cronbach’s alpha was.80 [25]; for the K-CERQ, it was.922 [38], and in this study, it was.805.

### Date collection and procedure

Data from the primary study [25], used for this secondary analysis, were collected between August 21 and September 11, 2020 during COVID-19 pandemic, via a web-based survey created using Naver Office’s online platform. Recruitment advertisements and survey links were posted on the university community website and Instagram to increase accessibility with minimal time and location restrictions. The advertisements contained information on the study’s purpose and procedures, eligibility, potential benefits and risks, the voluntary nature of participation, and a guarantee of anonymity. After reading the information, participants were asked to confirm their undergraduate status before clicking “I agree” to provide informed consent, after which the full questionnaire was displayed.

### Ethics statement

The primary study [28] used in this secondary analysis was approved by the Institutional Review Board of Dongguk University (DGU IRB 20200025) and conducted in compliance with the ethical standards outlined in the Declaration of Helsinki. Participants were informed of the study’s purpose, procedures, and confidentiality safeguards. Informed consent was obtained from all participants. It was also stated that 30 participants who completed the survey would be randomly selected to receive a digital gift voucher worth 5,000 KRW. Only those who consented to participate and provided e-mail addresses were included in the study. For this secondary analysis, data with personally identifiable information removed were used in compliance with the same ethical standards.

### Data analysis and covariates

Age, sex, current stress, and current disease (including mental disorders), known risk factors for NSSI [7,42–44] were included as covariates. To focus on stress related to psychological discomfort during COVID-19, current stress was assessed via the single-item: “Do you experience any stress currently during the COVID-19 pandemic?” (yes/no). Respondents who answered “yes” were also asked to freely describe specific sources of stress. Similarly, participants were asked whether they currently had any illnesses (yes/no), followed by an open-ended item for details.

Data were analyzed using SPSS Statistics version 25.0 (IBM Corp, Armonk, NY, USA), STATA 14.0 (TX, USA), and PROCESS macro 3.4 (https://www.processmacro.org/index.html). Descriptive statistics were used to analyze participant characteristics and main variables. Independent t-tests and chi-square tests were used to identify group differences in general characteristics and main variables between NSSI and non-NSSI groups, and between perceived and non-perceived stress within the NSSI group. Pearson’s correlation coefficients were used for bivariate analyses. Among the variables, only NSSI frequency did not satisfy the normality assumption (skewness = 2.630, kurtosis = 6.584) [45]. Previous studies have reported that suicide and NSSI data tend to be zero-inflated and overdispersed [40]. Therefore, zero-inflated negative binomial regression (ZINBR) was deemed appropriate [46] for predicting risk and preventive factors of NSSI. The dependent variable was the frequency of NSSI; participants with no NSSI experience had a score of zero (0 = zero counts). In this sample, 61.7% of participants reported zero NSSI frequency, indicating a zero-inflated distribution. Accordingly, ZINBR was used to address data heterogeneity caused by the overrepresentation of non-NSSI responses. Model fit was verified using the log-likelihood ratio and Vuong test. Finally, Hayes PROCESS macro Model 1 was employed to evaluate the moderating effect of CERS on the relationship between psychological discomfort and NSSI [47,48]. In addition, the Johnson–Neyman and pick-a-point methods were used to examine the moderating effect of CERS between psychological discomfort and NSSI. The regions of significance yielded statistical significance transitions within the observed range of moderators using the Johnson–Neyman method. The pick-a-point method was used to plot conditional effects for low

(mean-1 × SD), medium, and high (mean + 1 × SD) levels of CERS. Moreover, the 95% bias-corrected confidence interval from 5000 resamples was generated using the bias-corrected bootstrapping method. The number of bootstrapping sizes was 5000. Significant indirect effects were identified when the confidence interval did not include zero.

## Results

### NSSI and Non-NSSI group characteristics and stressful events during COVID-19

Of the total participants, 57 (30.3%) reported experiencing NSSI during the last six months (Table 1). Among the NSSI group, approximately 96.5% were female, with a mean age of 20.54 (standard deviation [SD] = 1.62; range = 18–27 years). A total of 56 participants (five men and 51 women), including 26 from the NSSI group (all female, 45.6%), reported experiencing stress due to quarrels and conflicts at home; relationship issues (e.g., breakups or quarrels); employment and future career anxiety; academic achievement; adaptation to new environments; restrictions on outdoor activities; and interpersonal relationship restrictions due to COVID-19. Seven participants reported having current diseases, including depression (n = 2), atopy (n = 2), hypothyroidism (n = 1), arthritis (n = 1), and chronic rhinitis (n = 1); all five participants with diseases in the NSSI group were female.

**Table 1:** Differences in participants’ general characteristics and main variables according to NSSI (N = 188).

	Non-NSSI group (n = 131), M ± SD or n (%)	NSSI group (n = 57), M ± SD or n (%)	χ^2^ or t (*p*)
Age	20.63 ± 1.91	20.54 ± 1.62	0.30 (.758)
Sex			8.90 (.002)
Male	27 (20.6)	2 (3.5)	
Female	104 (79.4)	55 (96.5)	
Current diseases	2	5	5.81 (.028)
Current stress	30	26	9.79 (.003)
K-SHI	–	2.24 ± 1.81	–
Psychological discomfort	13.36 ± 8.88	22.92 ± 8.32	−6.91 (<.000)
PCERS	70.77 ± 11.11	66.92 ± 12.47	2.10 (.037)
Acceptance	11.83 ± 2.37	12.05 ± 2.23	−0.57 (.566)
Putting into perspective	14.11 ± 2.96	13.54 ± 3.32	1.16 (.245)
Positive refocusing	13.95 ± 3.43	12.82 ± 3.96	1.97 (.050)
Refocusing on planning	16.19 ± 2.63	15.59 ± 3.46	1.28 (.199)
Positive reappraisal	14.67 ± 3.48	12.91 ± 4.23	2.98 (.003)
NCERS	43.78 ± 9.30	50.26 ± 9.80	−4.31 (.001)
Self-blame	11.73 ± 2.98	13.22 ± 3.94	−2.85 (.005)
Catastrophizing	8.69 ± 3.67	11.17 ± 4.28	−4.04 (<.001)
Blaming others	10.32 ± 3.21	11.42 ± 3.80	−2.03 (.043)
Rumination	13.03 ± 3.43	14.43 ± 3.41	−2.57 (.011)

Note. NSSI: non-suicidal self-injury; K-SHI: Korean version of the Self-Harm Inventory; PCERS: positive cognitive emotion regulation strategy; NCERS: negative cognitive emotion regulation strategy; NSSI group: K-SHI > 0; Current disease and stress: the number of respondents who answered “yes.”; % is row or column.

The mean K-SHI score in the NSSI group was 2.24 (SD = 1.81). The mean scores for psychological discomfort, PCERS, and NCERS in the NSSI group were 22.92 (SD = 8.32), 66.92 (SD = 12.47), and 50.26 (SD = 9.80), respectively.

Significant differences were observed in sex, current disease, and stress levels between the NSSI and non-NSSI groups. The NSSI group had significantly higher psychological discomfort (t = −6.91, *p* < .000), lower PCERS (t = 2.10, *p* = .037), and higher NCERS (t = −4.31, *p* = .001) scores than the non-NSSI group. Among PCERS subfactors, “refocusing on planning” and “positive reappraisal” were used more frequently in the non-NSSI group, whereas “refocusing on planning” and “putting into perspective” were used more frequently in the NSSI group. Both groups showed high use of the NCERS subfactors “rumination” and “self-blame”, however, the non-NSSI group showed significantly lower use (t = −2.57, *p* = .011, t = −2.85, *p* = .005).

### Methods of NSSI and endorsement rates by subjective awareness of NSSI

Among the 57 participants in the NSSI group, 46 responded that they had never attempted NSSI (non-perceived group), despite having engaged in NSSI during the last six months. This number is approximately four times higher than those who answered “yes” to the question, “Have you ever experienced NSSI?” (perceived group). In the perceived group, the mean age at which NSSI started was 14.36 years. Among all 57 NSSI participants, the most frequently endorsed NSSI methods were “hit yourself,” “overdosed,” and “banged your head,” in that order. The perceived group showed higher rates of methods such as overdosing, hitting, banging the head, scratching, and preventing wounds from healing compared to the non-perceived group. In the non-perceived group, behaviors such as overdosing, hitting, banging the head, and scratching were more common than mistreating or abusing oneself, being in abusive relationships, or preventing wounds from healing. Over the past six months, the perceived group employed an average of 3.9 NSSI methods, whereas the non-perceived group attempted approximately 1.85 methods (Table 2).

**Table 2:** Differences in frequency and methods of NSSI by subjective awareness of NSSI (Duplicate response).

K-SHI items	NSSI group (n = 57)	
	Perceived (n = 11) n (%)	Non-perceived (n = 46) n (%)
Age when NSSI first started	14.36 ± 2.11	–
K-SHI (within the last six months)	3.90 ± 1.75	1.84 ± 1.60
Overdosed	5 (45.5)	18 (39.1)
Cut	4 (36.4)	3 (6.5)
Burned	0 (0.0)	0 (0.0)
Hit	7 (63.6)	16 (34.8)
Banged the head	6 (54.5)	13 (28.3)
Scratched	6 (54.5)	10 (28.3)
Prevented wounds from healing	6 (54.5)	5 (10.9)
Setup in a relationship to be rejected	4 (36.4)	11 (23.9)
Engaged in emotionally abusive relationships	4 (36.4)	8 (17.4)
Engaged in sexually abusive relationships	1 (9.1)	1 (2.2)

Note. K-SHI, Korean version of the Self-Harm Inventory; NSSI, non-suicidal self-injury.

### Correlations among study variables

As shown in Table 3, NSSI was positively correlated with psychological discomfort (r = .363, *p* < .001) and NCERS (r = .269, *p* < .001), and negatively correlated with PCERS scores (r = −0.267, *p* < .001).

**Table 3:** Correlation among study variables.

	M ± SD	Range	1	2	3	4
1. Psychological discomfort	16.26 ± 9.74	0-39	1			
2. PCERS	69.61 ± 11.64	29-95	−.267^**^	1		
3. NCERS	45.75 ± 9.89	20-71	.402^**^	−.168^*^	1	
4. NSSI	0.68 ± 1.43	0-7	.363^**^	−.253^**^	.269^**^	1

Note. NSSI, non-suicidal self-injury; PCERS, positive cognitive emotion regulation strategy; NCERS, negative cognitive emotion regulation strategy.

### Predictors of NSSI

Using the ZINBR model, the likelihood-ratio test χ^2^ showed a significance level of <.001, indicating good model fit. Only statistically significant variables are presented in Table 4. In the count group, female sex (β = 1.78, *p* = .005) and PCERS (β = −0.02, *p* = .018) were significant predictors of NSSI, indicating that being female and having lower PCERS scores were correlated with increased NSSI frequency. NCERS was also a marginally significant predictor of increased NSSI frequency (β = 0.03, *p* = .018). In the logit group (participants reporting 0 NSSI frequency), psychological discomfort was a significant risk factor (β = −0.47, *p* = .043); higher psychological discomfort was associated with a higher likelihood of NSSI (Table 4). Other variables, including covariates, were not significantly associated with NSSI in either the count or logit groups.

**Table 4:** Zero-inflated negative binomial regression analysis for frequencies of NSSI (N = 188).

Model	Variables	Coefficient	95% CI	*p*
Count model	Female	1.78	−0.06–3.63	.005
	PCERS	−0.02	−0.05–-0.00	.018
Logit model	Psychological discomfort	−0.47	−0.92–-0.01	.043
Likelihood-ratio test χ^2^ (*p*)		12.76 (<.001)		
Vuong test z (*p*)		3.26 (<.001)		

Note. NSSI: non-suicidal self-injury; PCERS: positive cognitive emotion regulation strategy; NCERS: negative cognitive emotion regulation strategy; CI: confidence interval. Count model for persons who had experienced NSSI. Logit model for persons who had never experienced NSSI. Variables: Sex, Age, Current disease, Current stress, Psychological discomfort, PCERS, and NCERS. Dummy variables: Sex (female), Current disease (yes), and current stress (yes).

### Moderating effect of CERS

**Fig 2:**
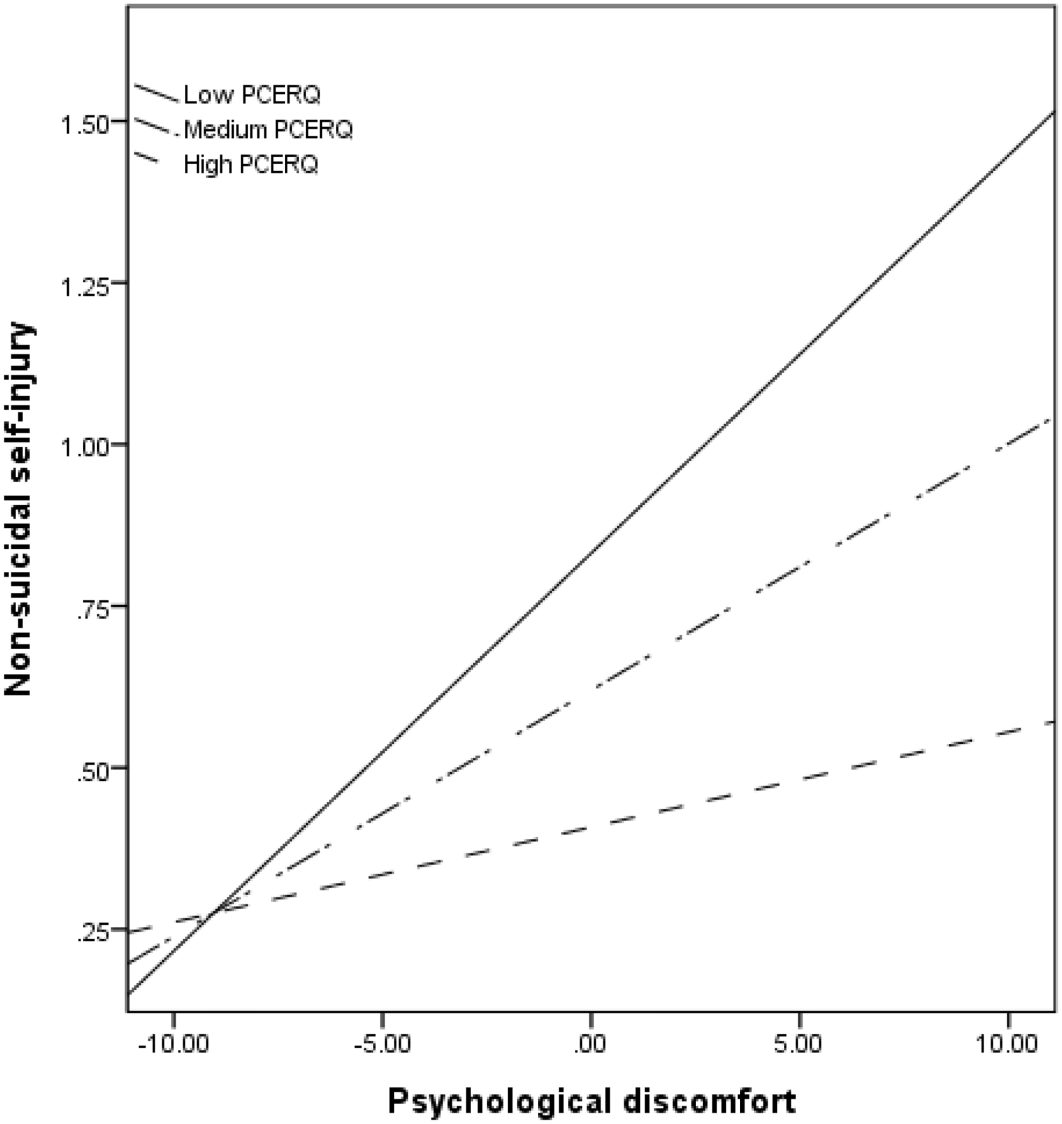
Moderating effects of PCERS between psychological discomfort and NSSI of undergraduate students (Low designates −1 SD and high designates +1 SD on the scale).

As shown in Table 5, psychological discomfort had a significant positive effect on NSSI (β = −0,04, *p* = < .001), supporting Hypothesis 1. Regarding Hypothesis 2, PCERS moderated the relationship between psychological discomfort and NSSI. There was a significant interaction effect between psychological discomfort and PCERS on NSSI (β = −0,00, *p* = .025). The conditional moderating effects of PCERS, calculated using the Johnson–Neyman method, indicated no significant effect for positive scores ≥ 6.468 (27.66%). As presented in Fig 2, the positive association between psychological discomfort and NSSI was significant at low (β = 0.06, t = 4.04, 95% CI [0.03, 0.09], *p* < .001) and medium (β = 0.04, t = 3.38, 95% CI [0.02, 0.06], *p* < .001) levels of PCERS. However, NSSI was not significant at the high level (β = 0.02, t = 0.95, 95% CI [−0.02, 0.04], *p* = .341) of PCERS. NCERS did not significantly moderate the relationship between psychological discomfort and NSSI (β = 0.02, t = 1.74, *p* = .083).

**Table 5:** Moderating effect of PCERS on the relationship between psychological discomfort and NSSI.

Outcome	Predictors	β (95% CI)	SE	*P*
NSSI	Constant	0.06 (−2.86–2.98)	1.48	.967
	Psychological discomfort	0.04 (0.02 - 0.06)	0.01	<.001
	PCERS	−0.02 (−0.04 - −0.00)	0.01	.039
	Psychological discomfort x PCERS	−0.00 (−0.00 - −0.00)	0.00	.025
F	6.234			
R2	0.195			<.001
△R2	0.023			.025

Note. NSSI: non-suicidal self-injury; PCERS: positive cognitive emotion regulation strategy; NCERS: negative cognitive emotion regulation strategy; CI: confidence interval; SE: standard error.

Model was adjusted for age, sex, current diseases and stress.

## Discussion

Approximately 30.3% of participants were found to have engaged in NSSI behaviors in the past six months, which is 1.2–1.8 times higher than previously reported rates [6,49]. Furthermore, 34.6% of female students had engaged in NSSI during this period, which is 2.76 times the previously reported incidence among female students (12.5%) [9]. In this study, 96.5% of the NSSI group were female, consistent with findings that females, including late adolescents, are more susceptible to NSSI than males [44,50,51]. The high incidence among women may be associated with stress [2,5,7] and illness [44], as reported in previous studies. All participants in the NSSI group who reported current stress and illness in this study were women. Stress especially affects NSSI among female students. However, NSSI itself may also become a source of stress [7]. Furthermore, participants identified severe interpersonal relationships due to COVID-19 as stressors, in addition to previously reported factors such as friendships, academic demands, and family problems [2,5]. Late adolescent females vulnerable to peer relationships often use negative emotion regulation strategies such as avoidance and rumination when experiencing daily distress, such as peer arguments [51–53], and are more likely to engage in NSSI to manage internal distress [51]. The COVID-19 crisis introduced unique stressors for undergraduates, such as uncertainty about the future, employment challenges, academic disruption, social isolation, and health-related concerns [14,53,54]. These factors have triggered psychological discomfort (e.g., depression and anxiety) among college students, with 71.26% reporting elevated stress and anxiety, and 18.04% reporting suicidal thoughts [54]. This study similarly found that the NSSI group experienced approximately 1.8 times more psychological discomfort than the non-NSSI group, indicating that the pandemic exacerbated both discomfort and NSSI scores. In addition, 8.8% of participants in the NSSI group had physical or mental health conditions such as atopic dermatitis and depression, supporting previous findings that such conditions are associated with NSSI [9,44,55].

The high incidence of NSSI in this study may also reflect the survey methodology. Forty-six participants in the NSSI group claimed no prior NSSI history, more than four times the number who reported a history. This aligns with previous reports that anonymous surveys [56] and self-report checklists (rather than single-item questions) yield higher NSSI disclosure rates [57]. This study also showed that participants used more than one NSSI method, consistent with Cipriano et al. (2017) [44]. Moderate methods (e.g., hitting and scratching) were more common than severe behaviors in both perceived and non-perceived groups. This may result from limited awareness of NSSI rather than flaws in the assessment method [58]. Hence, increasing awareness of problematic behaviors may promote healthy behaviors (e.g., seeking help in times of crisis) [59]. Therefore, students’ understanding of NSSI should be enhanced. School counselors and community care managers have only moderate NSSI knowledge and lack adequate NSSI-related education [60–62]. Therefore, policies should be developed to boost students’ understanding of NSSI and promote healthy behaviors such as seeking help during a crisis. Orientation or educational programs should introduce NSSI concepts, including the definition of NSSI and examples of problem behaviors, to all students early in college. In particular, participants in the perceived NSSI group began NSSI at a mean age of 14 years, and those in the non-perceived NSSI group likely also began during adolescence. As the delay in seeking help for adolescent self-harming ranges from one to two years [63], active counseling and intervention are needed as students enter college. Therefore, information about help-seeking resources, counseling hotlines, and campus counseling centers should be provided to all students through orientation programs, notices, and campaigns. Additionally, continuous education should be provided to health professionals in schools and communities, as well as training for gatekeepers. Future studies should investigate the association between NSSI and knowledge among undergraduate students.

The ZINBR model identified psychological discomfort as a risk factor for NSSI, increasing the likelihood of attempting NSSI even in the non-NSSI group. In other words, although there was no history of NSSI in the non-NSSI group, if psychological discomfort, such as depression or anxiety, increased, the probability of attempting NSSI also increased. This finding supports previous findings that psychological discomfort increases NSSI [3]. Thus, psychological discomfort should be considered when assessing risk factors in the non-NSSI group. In addition, the NCERS score was a marginally significant predictor of NSSI. The NSSI group utilized all NCERS strategies more frequently than the non-NSSI group. This supports previous results that rumination [24] and self-criticism [64] are more prevalent in the NSSI group. The PCERS score was also a predictor of NSSI. Therefore, it is necessary to focus on CERS in the NSSI group. This suggests that a differentiated preventive intervention strategy is required depending on whether a group has experienced NSSI.

In the present study, the incidence of NSSI significantly increased with psychological discomfort when PCERS scores were low or medium. Conversely, high PCERS scores can be interpreted as a preventive factor. This supports previous findings that PCERS can reduce psychological discomfort [25] and NSSI [3]. The non-NSSI group frequently experienced positive refocusing and reappraisal. Although statistically insignificant, the PCERS “acceptance” score was higher in the NSSI than in the non-NSSI group, which accords with previous results [26]. As Madjar et al. reported, the NSSI group tended to accept negative events, which in turn increased the likelihood of maladaptive coping behaviors [26]. Future studies should investigate whether acceptance is a valid PCERS sub-factor. Based on our findings, policies or psychoeducation should focus on increasing PCERS among undergraduate students with a history of NSSI, while interventions to lower psychological discomfort should target students without such a history. To achieve this, it is necessary to establish and apply strategies to enhance access to mental health resources on college campuses.

### Limitations

This study has several limitations. First, similar to other studies related to COVID-19 [15,54], we did not obtain information on individual experiences related to COVID-19 (infection or contact with infected individuals), as doing so could have negatively affected responses. The study used a cross-sectional design and lacked pandemic data, limiting causal interpretation. Longitudinal studies are required to address these limitations. Second, data on NSSI behaviors relied on sel-report and may be subject to recall bias. In addition, convenience sampling and the sex ratio, a factor influencing NSSI, was imbalanced, and only students with internet access could participate, which may limit generalizability.. Future studies should consider in-depth, short interviews and mixed-method approaches using more diverse and balanced samples. In addition, it is recommended that future research expands the “non-perceived” NSSI group to include possible cultural influences on self-recognition of self-injury, especially in Korea, where stigma around mental health and emotional expression may be stronger. Third, this study focused on only the cause of stress rather than the degree of stress that undergraduate students had recently experienced during the COVID-19 pandemic; therefore, a single item was used to assess stress. Single-item stress questions are widely used, and a single item on a Likert scale has been reported as a reliable and valid method for evaluating an individual’s stress [65,66]. However, in this study, a single item was answered with a “yes” or “no” to an open-ended question, which may have influenced the outcome. Considering that stress is one of the main variables affecting NSSI [5,7], future research should evaluate the degree of stress using validity tools along with its content. In addition, while several important covariates were controlled for in this study, we were unable to consider other covariates known to be associated with NSSI outcomes, such as childhood trauma [44], a prior history of NSSI [42], bullying [43], dating violence [2], and hopelessness [42,67]. These factors may have acted as confounding variables, potentially affecting the study outcomes. Finally, we did not measure the frequency of each NSSI behavior; thus, the severity of the NSSI could not be determined. Future studies should consider the duration of each type of NSSI.

### Strengths

Despite these limitations, this study offers several strengths. First, it confirmed that NSSI risk factors in the non-NSSI group significantly differed from those for increased NSSI in the NSSI group and presented evidence regarding the need to establish tailored preventive intervention strategies for different groups. Second, it expanded knowledge of NSSI predictors and confirmed the moderating effects of PCERS. Third, it confirmed the usefulness of the Symptom Checklist in measuring psychological states associated with NSSI, especially when rooted in trauma or stress. Finally, this study is relevant and timely, as it assessed NSSI among undergraduate students during the COVID-19 pandemic.
